# Variation in Neonatal Nutrition Practice and Implications: A Survey of Australia and New Zealand Neonatal Units

**DOI:** 10.3389/fnut.2021.642474

**Published:** 2021-08-02

**Authors:** Gemma McLeod, Shelley Farrent, Melissa Gilroy, Denise Page, Colleen J. Oliver, Fiona Richmond, Barbara E. Cormack

**Affiliations:** ^1^Neonatology, Child and Adolescent Health Service, Nedlands, WA, Australia; ^2^Faculty of Health and Medical Sciences, University of Western Australia, Perth, WA, Australia; ^3^Telethon Kids Institute, University of Western Australia, Perth, WA, Australia; ^4^Flinders Medical Centre, Bedford Park, SA, Australia; ^5^Mater Health Services, Brisbane, QLD, Australia; ^6^Mater Research Institute, University of Queensland, Brisbane, QLD, Australia; ^7^Royal Women's Hospital, Melbourne, VIC, Australia; ^8^Mercy Hospital for Women, Melbourne, VIC, Australia; ^9^Starship Children's Health, Auckland, New Zealand; ^10^Liggins Institute, University of Auckland, Auckland, New Zealand

**Keywords:** neonatology, enteral nutrition, parenteral nutrition, growth, surveys and questionnaires, dietitians and nutritionists, neonatal nutrition practice, standardized nutrition practice

## Abstract

**Background:** Significant global variation exists in neonatal nutrition practice, including in assigned milk composition values, donor milk usage, fortification regimens, probiotic choice and in methods used to calculate and report nutrition and growth outcomes, making it difficult to synthesize data to inform evidence-based, standardized nutritional care that has potential to improve neonatal outcomes. The Australasian Neonatal Dietitians' Network (ANDiN) conducted a survey to determine the degree to which neonatal nutritional care varies across Australia and New Zealand (A&NZ) and to highlight potential implications.

**Materials and Methods:** A two-part electronic neonatal nutritional survey was emailed to each ANDiN member (*n* = 50). Part-One was designed to examine individual dietetic practice; Part-Two examined site-specific nutrition policies and practices. Descriptive statistics were used to examine the distribution of responses.

**Results:** Survey response rate: 88%. Across 24 NICU sites, maximum fluid targets varied (150–180 mL.kg.d^−1^); macronutrient composition estimates for mothers' own(MOM) and donor (DM) milk varied (Energy (kcal.dL^−1^) MOM: 65–72; DM 69–72: Protein (g.dL^−1^): MOM: 1.0–1.5; DM: 0.8–1.3); pasteurized DM or unpasteurized peer-to-peer DM was not available in all units; milk fortification commenced at different rates and volumes; a range of energy values (kcal.g^−1^) for protein (3.8–4.0), fat (9.0–10.0), and carbohydrate (3.8–4.0) were used to calculate parenteral and enteral intakes; probiotic choice differed; and at least seven different preterm growth charts were employed to monitor growth.

**Discussion:** Our survey identifies variation in preterm nutrition practice across A&NZ of sufficient magnitude to impact nutrition interventions and neonatal outcomes. This presents an opportunity to use the unique skillset of neonatal dietitians to standardize practice, reduce uncertainty of neonatal care and improve the quality of neonatal research.

## Introduction

Significant global variation exists in neonatal nutrition practice, including in assigned milk composition values (1–4), donor milk usage (5), fortification regimens (6–9), nutrient supplementation (10–14), choice of strains and dosing of probiotics (15), and in methods used to calculate, assess and report nutritional intakes (16) and growth outcomes (17).

Implementation of evidence-based, standardized feeding guidelines has resulted in improved nutritional outcomes (18, 19) and lower rates of necrotizing enterocolitis (20) but even standardized nutrition practices vary between neonatal intensive care unit (NICU) sites and growth outcomes have been shown to differ across institutions (21).

Observational cohort or case-control study design has dominated preterm nutrition research. Much of this research has been underpowered and short-term in design. The inter-relationship between nutrition, growth, and neurodevelopmental outcomes reported in these studies can only be interpreted as associative, not causative and results are rarely conclusive (22–25). There is lack of good quality evidence upon which to base neonatal nutrition practice, leading to clinician opinion or consensus rather than gold standard evidence governing decisions around neonatal nutritional care (26).

Nutrition surveys are important tools as they facilitate a medium through which variations in nutrition practice can be identified, compared against peer and institutional practices, and assessed against existing evidence and expert recommendations.

Indeed, through a survey of neonatologists working in Regional Perinatal Centres (RPC), and using their database of mandatory reporting, the New York State (NYS) Perinatal Quality Collaborative noted marked variations in both nutrition practice across centers and in the incidence of extrauterine growth restriction (EUGR) among very preterm infants born within, or transferred to, their RPC within the first 48 h of life (27). Nutrition practices associated with EUGR included age at first and full enteral feeding, trophic feeding duration and volume, and duration of total parenteral nutrition. These findings were impetus for a state-wide quality improvement initiative to reduce practice variation that resulted in significant reduction in weight z-score change from birth to discharge (28).

An earlier survey of neonatologists in Australasian neonatal intensive care units found wide variation in clinical practice, identifying 50% of units without a written enteral feeding guideline and a lack of evidence supporting many nutrition practices (29).

A decade on, the Australasian Neonatal Dietetic Network (ANDiN) conducted a nutritional survey to determine the degree to which neonatal nutrition practices are standardized across Australia & New Zealand (A&NZ). We aim to use the survey results to improve standardization of dietetic practice, promote consensus on evidence-based practice, and inform the design of new research (26) to improve neonatal nutrition care and outcomes.

## Materials and Methods

The survey questions were predominantly multichoice with a large number of response options, including the option to provide a free text alternative answer, and to provide additional comments or further clarity about a response. The survey also included demographic, rating and ranking scale questions and was 'piloted' on a small focus group of ANDiN members.

A link to the 2-part, pre-tested electronic survey (Survey Monkey) was emailed to all ANDiN members (*n* = 50) working in neonatal units across A&NZ (*n* = 40).

Part 1 was designed to determine individual dietetic practice and targeted to individual, practicing neonatal dietitians; Part 2 was site-specific and designed to determine the nutrition policies and practices employed in neonatal units across A&NZ; one site-specific survey was completed per site.

Non-responders were sent two email reminders; the first, 4 weeks after recipients were mailed the invitation to participate and the link to the e-survey, and the second just prior to closure of the e-survey. For any non-response from an Australian and New Zealand Neonatal Network (ANZNN)-participating NICU, a further email was sent to a neonatologist to request the email address for the site dietitian or to confirm the non-existence of a neonatal dietitian at that site.

The e-Survey ran from July 16 2018 to October 10 2018. Responses were stored in a password protected online site. The Checklist for Reporting Results of Internet E-Surveys (CHERRIES) statement guideline was followed for reporting the survey results.

Descriptive statistics were used to examine the distribution of responses and percentages were calculated for categorical variables.

Ethics approval for conducting the survey was obtained through the Auckland District Health Board Research Review Committee (ID A+8146) and each survey responder was advised that online submission of their survey response signaled their consent to participate in the study.

## Results

Forty-four (88%) dietitians [Australian: *n* = 34 (77%); NZ: *n* = 10 (23%)], working across 40 hospital sites responded to Part 1 of the electronic survey. Twenty-seven of the dietitians worked in 25 ANZNN-participating Level 3 Units and another 10 worked across 9 Level 2 ANZNN Units.

Thirty-five from a possible 40 (88%) A&NZ site-specific responses were received for Part 2 of the e-Survey, including 33 ANZNN-participating Units (NICU *n* = 24; Level 2 *n* = 9). One response covered two ANZNN NICU sites.

## Part 1–Individual Neonatal Dietetic Practice

### Energy Values for Calculating Parenteral and Enteral Intakes

The energy values used by dietitians to calculate parenteral dextrose and lipid intakes ranged from 3.4 kcal (14.2 kJ).g^−1^ to 4 kcal (16.7 kJ).g^−1^, and 9 kcal (37.7 kJ).g^−1^ to 10 kcal (41.8 kJ).g^−1^, respectively. The vast majority of dietitians used the energy value of 4 kcal (16.7 kJ).g^−1^ to calculate enteral protein and carbohydrate intakes and all dietitians used the value of 9 kcal (37.7 kJ).g^−1^ for calculating enteral fat intake (Table 1).

**Table 1:** Survey–part one questions and responses.

**Question**	**Response rate n/44 (%)**	**Response**	**No. of respondents per answer**
**Part One: Completed by dietitians working in neonatal units** ***n*** **= 44**			
What energy value (kcal (kJ).dL^−1^) do you use for mature preterm breast milk for clinical work?	40 (91%)	65 (268)	1
		66 (276)	14
		67 (280)	6
		68 (284)	1
		70 (293)	10
		72 (301)	8
What protein value (g.dL^−1^) do you use for mature preterm breast milk for clinical work?	40 (91%)	1.00	5
		1.03	4
		1.20	9
		1.26	1
		1.27	5
		1.3 (rounding from 1.27)	13
		1.36	1
		1.4	1
		1.5	1
What reference do you use for preterm breast milk macronutrient composition?	38 (86%)	Boyce et al. (1)	14
		U.S Department of Agriculture (USDA) data	5
		NHMRC NRV (36)	3
		StRONNG checklist 2016 (16)	1
		Royal Children's Hospital (RCH)	1
		FoodWorks™ Xyris Software	5
		Schanler et al. (30)	1
		Koletzko et al. (31)	1
		Neonatal and Infant Handbook	6
		Unsure	1
What energy value [kcal (kJ).dL^−1^] do you use for donor milk?	38 (86%)	65 (268)	1
		66 (276)	4
		67 (280)	2
		68 (284)	1
		69 (289)	4
		72 (301)	3
		Not applicable	23
What protein value (g.dL^−1^) do you use for donor milk?	38 (86%)	0.8	4
		0.9	1
		1.0	3
		1.1	1
		1.27	1
		1.3	4
		1.36	1
		Not applicable	23
What reference do you use for donor breast milk macronutrient composition?	26 (59%)	Wojcik et al. (32)	2
		NHMRC (33), with modification for protein	1
		Boyce et al. (1)	2
		FoodWorks™	1
		USDA data	2
		Cooper et al. (34)	1
		Personal communication donor milk banks	2
		Unknown source	2
		Not applicable	13
Do you change the figures you use for energy and protein in breast milk, depending on the age of the baby for clinical work (e.g., colostrum vs. transitional vs. mature milk)?	40 (91%)	Yes	12
		Sometimes	3
		Never	25
How do you calculate weight gain (g.kg^−1^d^−1^) in the clinical setting?	39 (87%)	Net weight gain over the time interval divided by the time interval and the average of birth weight and weight at day x	13
		Net weight gain over time interval divided by time interval and current weight	9
		Exponential method Patel et al. (35, 36)	7
		Other–method not reported	2
		Not applicable	8
How do you calculate weight gain (g.kg^−1^d^−1^) in the research setting?	40 (91%)	Net weight gain over the time interval divided by the time interval and the average of birth weight and weight at day x	6
		Net weight gain over time interval divided by time interval and current weight	2
		Exponential method Patel et al. (35, 36)	8
		Other–method not reported	1
		Not applicable	23
What value do you use for [kcal (kJ).g^−1^] enteral protein in clinical work?	40 (91%)	3.8 (15.9)	4
		4.0 (16.7)	29
		Other value not specified	1
		Not applicable	6
What value do you use for enteral carbohydrate [kcal (kJ).g^−1^] in clinical work?	40 (91%)	3.8 (15.9)	6
		4.0 (16.7)	27
		Not applicable	7
What value do you use for enteral fat [kcal (kJ).g^−1^] in clinical work?	39 (89%)	9 (37.7)	32
		Not applicable	7
What value do you use for parenteral dextrose [kcal (kJ).g^−1^] in clinical work?	38 (86%)	3.4 (14.2)	6
		3.8 (15.9)	7
		4.0 (16.7)	9
		Not applicable	16
What value do you use for parenteral lipid [kcal (kJ).g^−1^] in clinical work?	40 (91%)	9 (37.7)	12
		10 (41.8)	11
		Not applicable	14
Do you ever prescribe commercial breastmilk fortifier for infants at hospital discharge?	40 (91%)	Once a week to once a month	2
		Less than once a month	9
		Never	26
		Never, but medical team may sometimes	3
Australian dietitians only. Do you ever prescribe Post Discharge Formula (specifically designed for preterm infants post-discharge from hospital) for formula fed babies on discharge from hospital?	32 (94%)	Sometimes (<1 month)	14
		Never	15
		No, concentrate term formula	2
		Not applicable	1

### Macronutrient Composition of Preterm and Donor Milk

The assumed macronutrient content of preterm breast milk was derived from a variety of sources and varied considerably across units. Protein content of mature preterm milk ranged from 1.0 to 1.5 g.dL^−1^, with 72% of dietitians using a value between 1.2 and 1.3 g.dL^−1^; energy content ranged from 66 kcal (276 kJ).dL^−1^ to 72 kcal (301 kJ).dL^−1^, with 50% of dietitians using a value between 66 kcal (276 kJ).dL^−1^ and 67 kcal (280 kJ).dL^−1^. Thirty-eight percent of dietitians changed assumed milk values according to day of expression, to reflect colostrum, transitional or mature milk. One third of dietitians used the values recommended in a recent systematic review of preterm human milk composition to estimate nutritional intakes from preterm breast milk, while 18% were unsure of the reference underpinning their practice (1) (Table 1).

The assumed values for protein and energy content of donor milk used by the fifteen dietitians working in neonatal units with access to donor milk ranged from 0.8 to 1.36 g.dL^−1^ and 65 kcal (272 kJ).dL^−1^) and 72 kcal (301 kJ).dL^−1^) and were derived from at least eight different sources, including local unpublished data (Table 1).

### Calculation of Weight Gain (g/kg/d)

Seventy-nine percent of the dietitians responding to this question calculated rate of weight gain in the clinical setting and most (42%) used the equation “net weight gain over the time interval, divided by the time interval and the average of birth weight and weight at day x.” Others (29%) calculated “net weight gain over the time interval divided by the time interval and current weight.” Only 23% of dietitians employed Patel's exponential method (35, 36) in the clinical setting (Table 1).

Of the dietitians (42%) who calculated rate of weight gain for research purposes, 47% used Patel's exponential method (35, 36), whilst 35% of dietitians calculated ‘net weight gain over the time interval, divided by the time interval and the average of birth weight and weight at day x’ (Table 1).

## Part 2–Site-Specific Nutrition Practices

### Target Feed Volumes

The range of target feeding volumes varied across sites, and was slightly lower for extremely low birth weight infants (birthweight <1,000 g; 150–180 mL.kg^−1^.d^−1^) than for very low birth weight infants (birthweight <1,500 g; 160–190 mL.kg^−1^.d^−1^). More than 40% of sites targeted 180 mL.kg^−1^.d^−1^ for non-fluid restricted preterm infants born <1,500 g. Maximum feeding rates ranged from 180 to 210 mL.kg^−1^.d^−1^ (Table 2).

**Table 2:** Survey-part two questions and responses.

**Question**	**Response rate n (%)**	**Response**	**No. of respondents per answer**						
**Part Two: One survey only completed per neonatal unit (** ***n*** **= 35 units)**									
Which country is your hospital in?	35 (100%)	Australia	25						
		New Zealand	10						
Does your unit have a written policy on the following?	28 (80%)	Screening criteria for dietitian referral	11						
		Enteral feeding	28						
		Intravenous feeding	25						
		Withholding feeds	14						
		Gastric aspirate volumes	14						
		Vitamin and mineral supplementation (inpatient)	24						
Is your unit a Baby Friendly Hospital?	34 (97%)	Yes	24						
		No	7						
		Unsure	3						
Which growth chart does your unit use for preterm infants?	34 (87%)	UK-WHO	7						
		Fenton 2003	2						
		Fenton 2007	1						
		Fenton 2013	17						
		Beeby 1996	1						
		NICUS (Kitchen 1983)	2						
		Intergrowth 21	1						
		Unsure	2						
		None	1						
Which growth charts does your unit use for term infants?	34 (97%)	WHO/UK-WHO	32						
		CDC	2						
What is the usual standard target feed volume (mL.kg^−1^.d^−1^) for ELBW infants (on standard fortification) when the infant is not fluid restricted?	34 (97%)	150	1						
		160	7						
		160–170	6						
		180	13						
		Unsure	4						
		Not applicable in our unit	1						
		No target	2						
What is the usual standard target feed volume (mL.kg^−1^.d^−1^) for VLBW infants (on standard fortification) when the infant is not fluid restricted?	34 (97%)	160	8						
		160–170	7						
		180	13						
		Unsure	3						
		No target	3						
What is the maximum feed volume (mL.kg^−1^.d^−1^) used in your Unit?	34 (97%)	180	10						
		190	3						
		200	14						
		210	2						
		Not applicable	5						
What brand of preterm formula is used in your unit?	34 (97%)	Brand A	18						
		(2.9 g protein.dL^−1^)							
		Brand B							
		(2.6 g protein.dL^−1^)	11						
		Brand C							
		(2.69 g protein.dL^−1^)	3						
		Don't use preterm formula	1						
How is the brand of preterm formula chosen in your unit? (Rank in order of importance)	32 (91%)	Nutritional composition	30						
		Osmolality	25						
		Tender product/standard feed that can be used	24						
		Volume in bottle	22						
		Same as nearby tertiary unit	22						
		Cost	22						
		Historical practice	21						
What brand of human milk fortifier is used in your unit?	34 (97%)	Brand A	1						
		(1.0 g protein.dL^−1^)							
		Brand B	31						
		(1.44 g protein.dL^−1^)							
		Brand C	3						
		(1.1 g protein.dL^−1^)							
How is the brand of fortifier chosen in your unit? Rank in order of importance.	30 (86%)	Nutritional composition	29						
		Volume of EBM the pack is added to	24						
		Osmolality	24						
		Tender/Standard feed that can be used	22						
		Mixability	22						
		Ease of pack opening	22						
		Historical practice	22						
If you chose nutritional composition or osmolality, rank in order of importance.	30 (86%)	Protein content	30						
		Osmolality	28						
		Iron content	28						
Where is breastmilk fortifier added to breastmilk in you Unit?	33 (94%)	Bedside	22						
		Dedicated milk room	8						
		Medication/formula bench/kitchenette in unit	3						
What is the maximum refrigeration time that milk can be stored in a refrigerator after fortifier has been added?	34 (97%)	Must be used immediately	3						
		4 h	14						
		6 h	1						
		8 h	4						
		12 h	2						
		24 h	10						
When do you start adding fortifier to breast milk, as per unit policy?	33 (94%)	Feed volume of 5 mL	6						
		Enteral feeds reach 80 mL.kg^−1^.d^−1^	2						
		Enteral feeds reach 90 mL.kg^−1^.d^−1^	1						
		Enteral feeds reach 100 mL.kg^−1^.d^−1^	7						
		Enteral feeds reach 120 mL.kg^−1^.d^−1^	2						
		After full enteral feeds are tolerated	6						
		Only if infant is growing poorly	6						
		Only added on dietetic recommendation, if infant is growing poorly	1						
		Doctors discretion	1						
		Unsure	1						
At what strength is breast milk fortifier routinely commenced in your Unit?	34 (97%)	Quarter strength or less	1						
		½ strength	13						
		Full strength	19						
		Unsure	1						
Is fortifier ever used beyond full strength in your Unit?	34 (97%)		Never	Rarely (once/y)	Occasionally (4 times/y)	Frequently (1/mo)	Routinely (daily or weekly)	Total	Weighted average
		1 ¼ str	28	2	4	0	0	34	1.29
		1 ½ str	29	1	2	0	0	32	1.16
		Double str	32	0	0	0	0	0	1
Are other modular supplements used in your Unit for infants?	34 (97%)		Never	Rarely (once/y)	Occasionally (4 times/y)	Frequently (1/mo)	Routinely (daily or weekly)	Total	Weighted average
		Glucose polymer	9	9	13	1	1	33	2.27
		Duocal	16	5	8	1	1	31	1.9
		Beneprotein	19	1	6	2	2	30	1.9
		Protifar	20	1	6	2	2	31	1.87
		Calogen	7	4	10	10	2	33	2.88
		Liquigen	14	5	6	2	1	28	1.96
		MCT oil	20	5	2	1	0	28	1.43
		Intravenous Amino Acids	24	3	0	0	1	28	1.25
		Infant formula powder	10	4	10	6	4	34	2.71
Are probiotics routinely provided for most infants in your Unit?	34 (97%)	Yes	23						
		No	8						
		Unsure	2						
If yes, what strain/s & dose is used	22 (96%)								
		Multi-strainLactobacillus acidophilus, Bifidobacterium infantis and Bifidobacterium bifidum;	0.16 mL BD						
		Lactobacillus GG	Unspecified dose						
		Bifidobacterium infantis and Lactobacillus acidophilus.	<750 g: $\frac{1}{4}$ capsule BD; 750 g – 1500 g: $\frac{1}{2}$ capsule BD >1500 g: 1 capsule BD Cease at 36 week and 2 kg.						
		Bifidobacterium breve M-16V	For infants <35 week gestation: 1 mL daily if minimal enteral feeds <50 mL.kg^−1^ 1 mL BD if minimal enteral feeds >50 mL.kg^−1^;						
Is food thickener used in your unit for preterm infants, and if so, what is used?	34 (97%)	Yes, Carob Bean Gum	24						
		Yes, rare, (Guar Gum	1						
		No	6						
		Unsure, rare, type not specified	3						
Is food thickener used as a gel for breast feeding preterm infants in your unit?	33 (94%)	Yes	5						
		No	27						
		Unsure	1						
Is donor breast milk available in your unit?	33 (94%)	No	15						
		Yes, via screened mother to mother, unpasteurised	7						
		Yes, via breast milk bank on site	3						
		Yes, via breast milk bank from another site (pasteurized	2						
		Yes, via breast milk bank purchased from another site	6						
If you use donor milk, is it routinely supplemented with modular supplements as well as breast milk fortifier? And if so, with what?	18 (100%)	No	13						
		Yes, Human milk fortifier +/- protein powder	5						

### Donor Milk, Milk Fortification and Food Thickener

Fifteen (45%) of the 33 sites responding to the question relating to donor milk access reported having none. Access to unpasteurized donor milk via screened mother to mother donations was available in seven (39%) of the 18 sites that reported using donor milk. The remaining 11 sites (61%) accessed donor milk through milk banks; eight (73%) of these purchased milk from a milk bank external to their own health-care facility and three (27%) accessed banked donor milk directly from their own respective on-site milk banks (Table 2).

The method and criteria for fortifying breast milk differed across sites. A little over 50% of sites fortified milk on the attainment of a specific volume intake per kilogram of body weight. However, there were a considerable range of acceptable intakes for commencing fortification across sites (80 up to 150 mL.kg^−1^.d^−1^). Other sites set a minimum feed volume of 5 mL as the criteria for commencing fortifier (18%) or fortified only if an infant demonstrated poor growth (18%). Of the 34 sites (97%) that provided a response, 56% commenced fortifier at full strength and 38% at half strength and most units (82%) did not use human milk fortifier to fortify milk beyond the strength directed by the manufacturer. Routine use of modular supplements to fortify breast milk, such as glucose polymer, protein powder or fat emulsions, was rare. Five (28%) of the 18 sites with access to donor milk routinely used protein powder in addition to human milk fortifier to fortify the donated milk. Breast milk was more commonly fortified at the bedside (67%) or in a dedicated milk room (24%). The maximum refrigeration time that fortified milk was stored following preparation varied from 0 to 24 h (Table 2).

Only 18% of respondents stated that their site did not use food thickener; in the majority (71%), carob bean gum thickener was the preferred choice and a small proportion of units (15%) used food thickener to make a gel for breastfeeding infants (Table 2).

### Choice of Preterm Formula and Human Milk Fortifier

**Figure 1:**
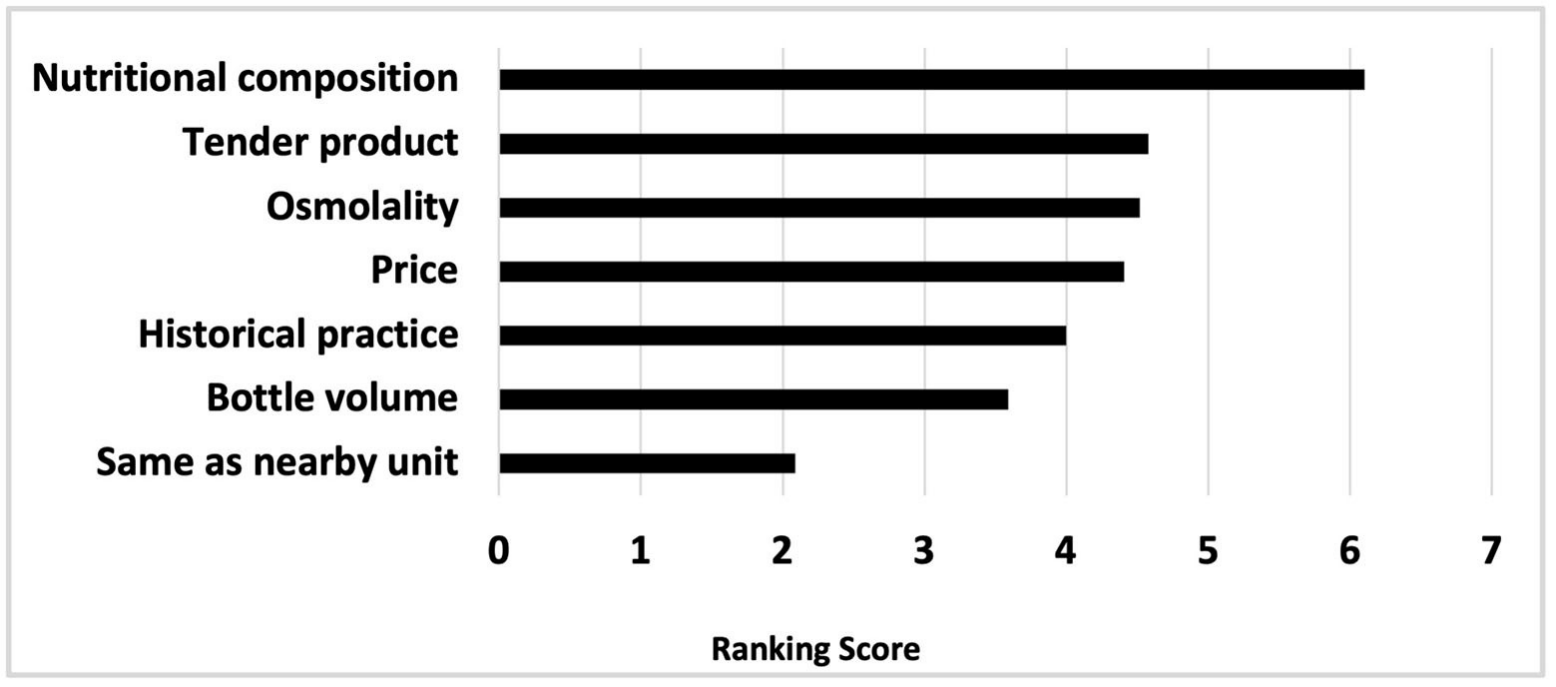
Reasons guiding preterm formula choice, ranked in order of importance.

**Figure 2:**
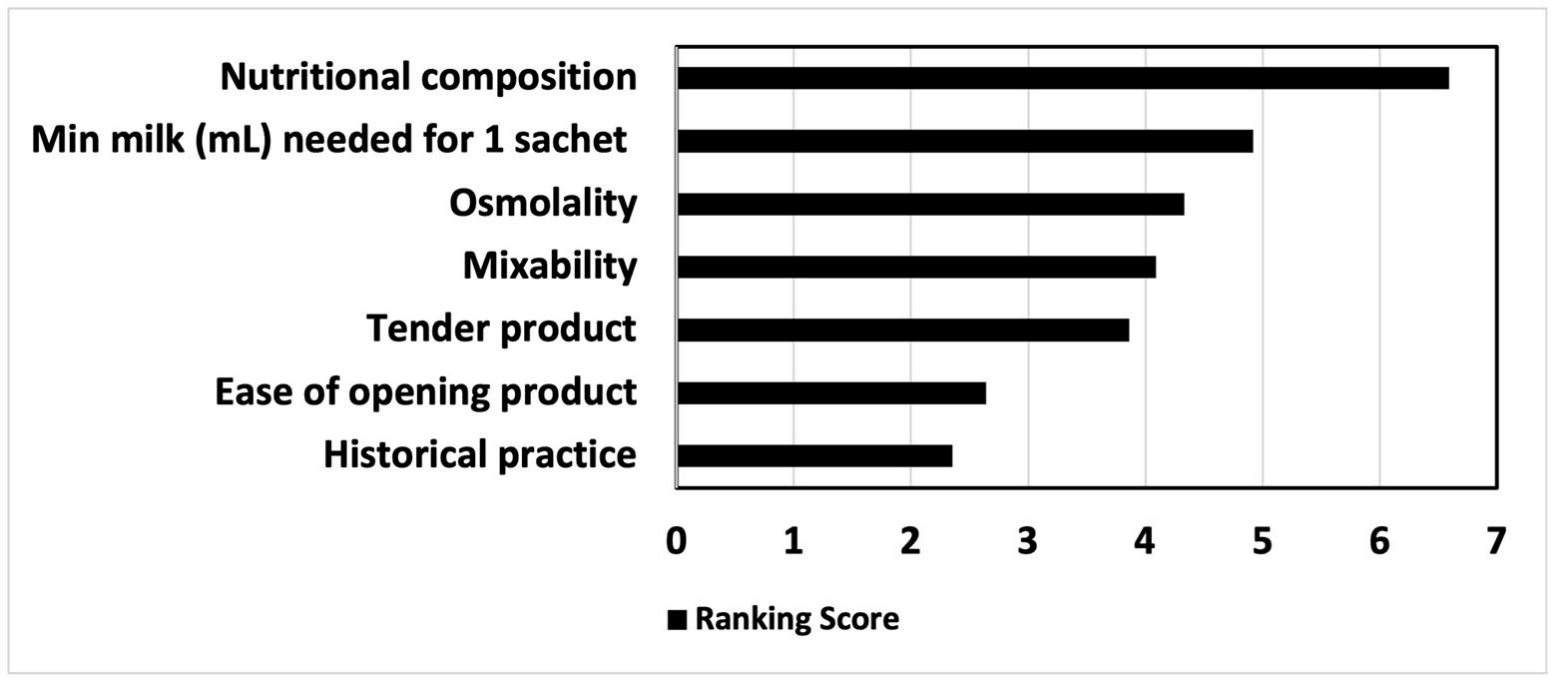
Reasons guiding human milk fortifier choice, ranked in order of importance.

**Figure 3:**
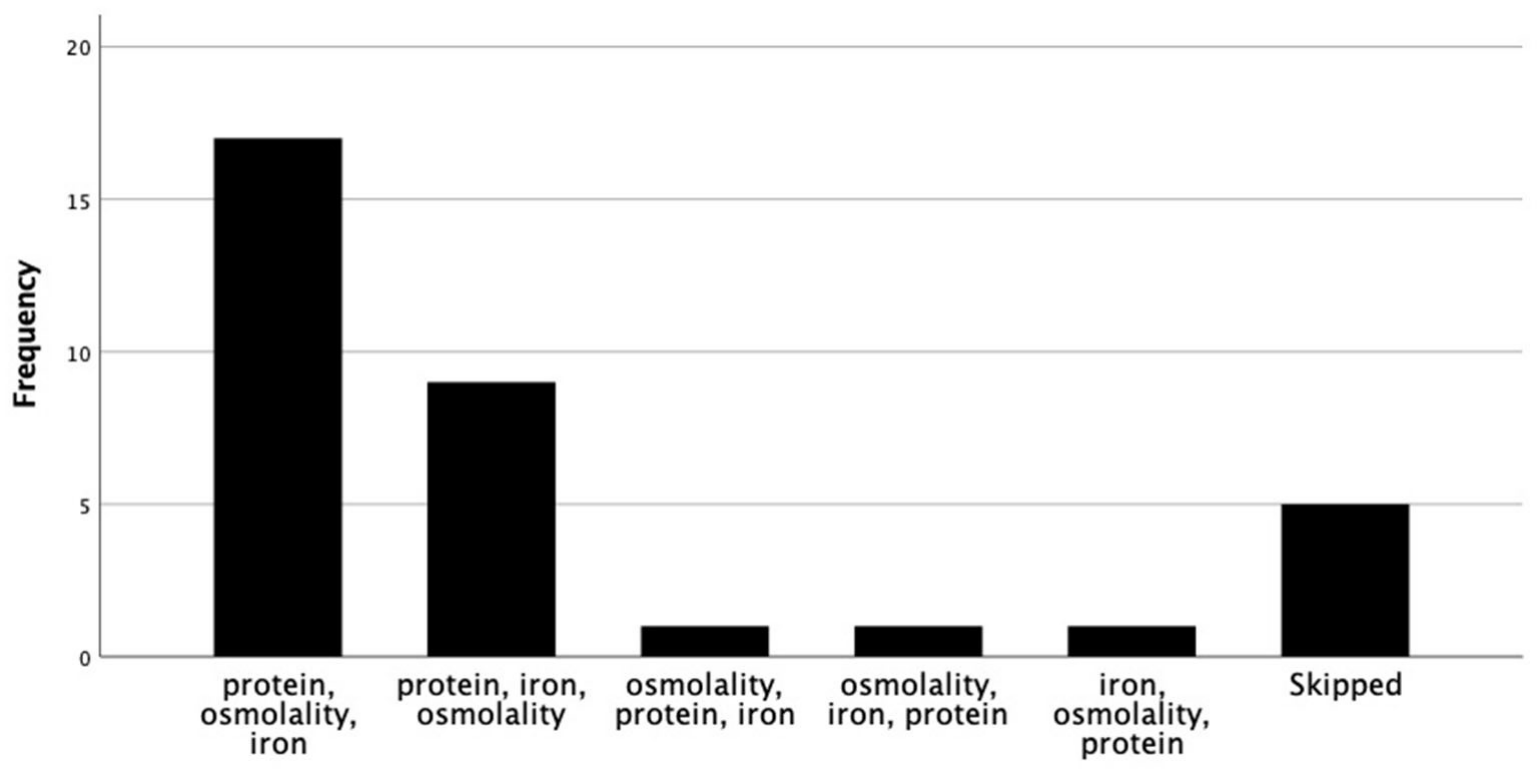
Nutritional factors ranked in order of importance, when nutrition composition guides fortifier choice.

Ranked in order of importance, the factors guiding choice of preterm formula and human milk fortifier are depicted in Figures 1, 2, respectively. For most, when nutritional composition guides choice, protein, osmolality and then iron are ranked as most important considerations (Figure 3).

### Probiotics

Probiotics are routinely used by at least 68% of respondent sites but different brands with various bacterial strains are in use and dosing regimens differ across sites (Table 2).

### Choice of Growth Charts

At least seven different growth charts, each derived from different sets of cross-sectional data, are employed across neonatal sites to monitor preterm growth (Tables 3, 4). Of the 34 sites (97%) using growth charts, the Fenton 2013 charts (40) are used by the majority (50%), while 21% of units are using the UK-WHO growth charts (39) and one site is using the Intergrowth-21 chart (Table 2).

Table 3Size-at-birth growth curves.

**Table:** 

**Growth charts**		**Kitchen et al. (37)**	**Beeby et al. (38)**	**UK-WHO (39)**	**Fenton- (40)**	**INTERGROWTH-21 very preterm size at birth reference charts (41) and Newborn baby size International Standards (42)**
Methodology		Pooled birth data from two datasets	Pooled birth data from two datasets	Pooled birth data from five data sets	Systematic review, selection and meta-analysis of 6 data sets	Multi-center, multi-cultural, multi-country population-based prospective study
Data collection time-period		1977–1979	1990–1995 (KGV) 1982–1989 (MDC)	1983–1993	1991–2007	2009–2014
Gestational age range (week)						
	Weight	24–42	20–42	23–42	22–50	24–42
	Length	25–42	<35	26–42	23–50	24–42
	Head Circ	25–42	<35	23–42	23–50	24–42
Age plotting		Completed weeks	Completed weeks	Completed weeks	Actual age	Completed weeks
GA assessment method		Ultrasound before week 20 or1st day of LMP	Verification of GA for MDC data not possible; Verification of GA for KGV data based on 1st trimester ultrasound or on basis of LMP when early ultrasound available.	Mixed –maternal dates confirmed by early ultrasound, clinician assessment, not specified	Mixed – early ultrasound, maternal dates, clinician assessment	Verification based on GA for MDC and confirmatory ultrasound dating scan.
Location		Melbourne, Australia. Livebirths at RWH in 1979 and infants born <35 week in 1977–1978Data of infants born 24–29 week in Queen Victoria Medical Centre (Kitchen et al. 63) also included.	New South Wales, Australia Midwives Data Collection–birthweights of live and still births (MDC); King George V(KGV) obstetric-neonatal data – birthweights, head circumference & length	United Kingdom, excluded ‘non-white’ participants.	Australia, Scotland, United States of America, Canada, Germany, Italy	Brazil, Italy, United Kingdom, United States of America, India, Kenya, China, Oman
Participant selection		No	No	No	No	>33 week: original Newborn Size Standards, incorporating measurements from neonates without major complications or ultrasound evidence of fetal growth restriction (FGR), who were born to mothers without FGR risk factors; For infants born ≤ 32 week GA, born of mothers with some FGR risk factors, but not smoking or obesity)
Measurement standardization		No	No	No	No	Yes

**Table:** 

Sample size	Gest week	<27^+6^	28^+0−^31^+6^	32^+0−^36^+6^	<27^+6^	28^+0−^31^+6^	32–36^+6^	<27^+6^	28^+0−^31^+6^	32^+0−^36^+6^	<27^+6^	28^+0−^31^+6^	32^+0−^36^+6^	<27^+6^	28^+0−^31^+6^	32^+0−^32^+6^	33^+0−^36^+6^
	Weight	252	230	265	1,039	1,956	17,930	706	1,437	426	22,942	44,472	192,881	82	203	97	1,022
	Length	196	215	232	139	236	1,423	49	180	62	9,605	20,211	120,214	63	186	94	1,014
	Head	108	170	165	388	942	2,247	325	760	52	9,605	20,211	120,214	76	173	95	1,016

**Table:** 

Centile lines		10th, 50th,and 90th	3rd, 10th, 25th, 50th, 75th, 90th, and 97th.	0.4th 2nd, 9th, 25th, 50th, 75th, 91st, 98th, and 99.6th	3rd, 10th, 50th, 90th, and 97th	3rd, 10th, 50th, 90th, and 97th
z-scores		No, Means, SD available	No, Means, SD available	Available	Available	Available
Gender specific		Not gender specific	Combined gender <30 week, then gender specific	Gender specific	Gender specific	Gender specific

**Table 4:** Key features of INTERGROWTH-21st postnatal preterm growth standard (43).

• 201 participants (99 males; 102 females) ◦ Brazil 36 (18%) ◦ China 18 (9%) ◦ India 3 (15%) ◦ Italy 24 (12%) ◦ Kenya 30 (15%) ◦ Oman 30 (15%) ◦ UK 22 (11%) ◦ USA 10 (5%)	
• Mean (SD; range) gestational age of participants 35.5 (1.7)	
<27 week 27–32 week 33 week 34– 35 week 36-<37 week	No data *n* = 12 (6%) *n* = 16 (8%) *n* = 68 (34%) *n* = 105 (52%)
• Mean weight of infants was 2,452 (SD 519) g.	
• Three measurements only taken in the first month of life, at birth, 2 and 4 week.	
• Postnatal nutritional intake not specified, quantified nor necessarily optimized.	
• Curves represent the data in completed weeks and do not harmonize with the WHO charts until 14 weeks post corrected term.	

A minimum of three different term growth charts are in use across sites (*n* = 34), but in the vast majority (94%), WHO/UK-WHO growth charts are the preferred tools for assessing the growth of term infants (Table 2).

Survey results discussing variations in vitamin supplementation regimens (44), dietetic resourcing and written nutrition policies (45) have been reported elsewhere.

## Discussion

Our ANDiN survey suggests that considerable variation in nutrition practice continues across neonatal units in A&NZ, in particular with respect to assigned milk composition values, timing and method of milk fortification, use of donor milk, type and brand of milk additives, targeted feed volumes, strains and dosing of prescribed probiotics, choice of growth charts and in methods used for calculating nutrition intakes and growth velocity (Tables 1, 2).

### Milk Composition and Fortification

Macronutrient milk composition, particularly lipid concentration, is influenced by stage of lactation, gestational age, maternal diet, and parity and there is significant diurnal and inter-feed variation (46). Determining an estimate of milk composition and energy content can be labor intensive, time-consuming and costly (7), and among studies, the integrity of the measurement can be confounded by variations in milk sampling strategies (47) and by the analytical methods employed in its determination (48, 49). For example, the gold standard sampling method for lipid analysis and measuring the energy content of milk is complete 24-h collection, whereas foremilk, mid-feed or hindmilk samples are adequate for measuring protein, lactose and oligosaccharides. Further, each energy-yielding macronutrient can be quantified using many different methods, each with its own limitations and degrees of precision and accuracy, and the energy content of milk can be estimated as either metabolizable or gross energy. The inherent differences in the various methods used to analyze each energy-yielding component of milk may result in discrepancies for comparison of absolute concentration of the different nutrients among studies. It is important that the implications of the different milk sampling strategies and the inherent differences and limitations of various analytical methods are understood and considered when designing research methodology and when applying milk composition values in the clinical setting.

Since 2014, at least four systematic reviews and two meta-analyzes on preterm human milk composition have been published (1–4). These reviews have included studies conducted over a period of decades. Each review has used different criteria for study inclusion and there is overlap of studies included across reviews. Critical analysis of the analytical methods used in the included studies has not been attempted by all reviewers.

Our survey identified that assumed rather than measured macronutrient values are more often employed in neonatal units across A&NZ to calculate estimated nutrition intakes and there is still a 50% variation between the lowest (1.0 g.dL^−1^) and highest (1.5 g.dL^−1^) assumed values used to quantify the protein content of preterm milk. The most commonly used value is sourced from a recent systematic review (1.27 g.dL^−1^), and reflects the median protein content of mature preterm milk expressed during weeks 2–8 of lactation from studies employing relatively robust analyzes and correcting for non-protein nitrogen (1).

Calculation of preterm nutrition intakes is based on a measured or assumed native composition of breast milk, which is known to vary between and within mothers, and according to whether the milk is fresh or frozen, and whether it is mother's own or raw or pasteurized donor milk. The various conversion factors employed to calculate the energy contribution from the macronutrients in the milk, as well as the nutrition profiles of the products employed in its fortification, also contribute to variations in how nutrition intakes are calculated.

**Figure 4:**
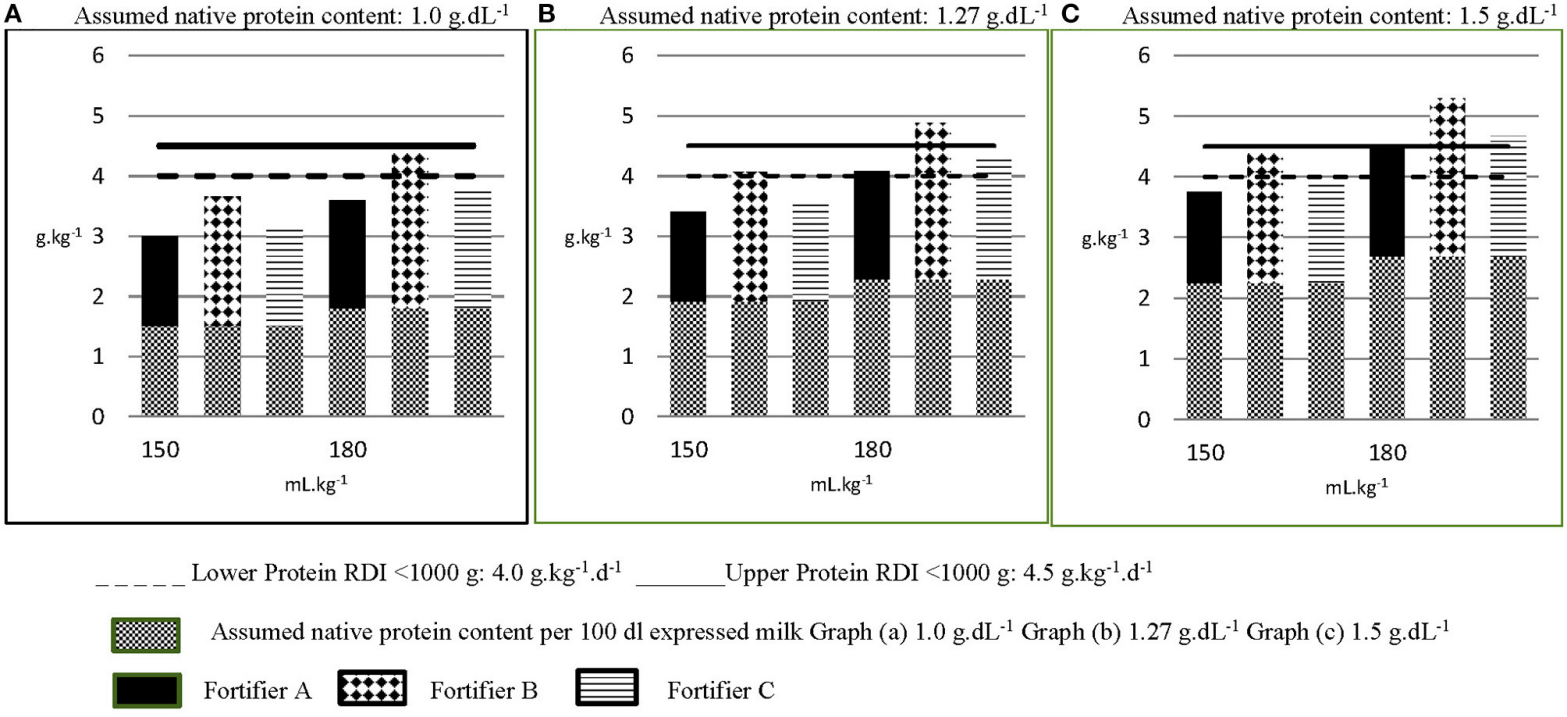
Calculated estimated protein intakes from different fortified milk feeds. The native protein content per 100 mL of milk feeds in Graphs A–C are 1.0 g, 1.27 g and 1.5 g, respectively, representing the lowest, most common and highest assigned values in use across A&NZ, as reported in the survey. Each graph **(A–C)** depicts three different fortified milk feeds fed at 150 mL.kg^−1^.d^−1^ and 180 mL.kg^−1^.d^−1^. The protein content of each milk feed is increased from its assumed native content, according to the protein content of the fortifier added (per 100 mL expressed milk: Fortifier A: 1.0 g protein; Fortifier B: 1.44 g; Fortifier C: 1.1 g). Graph **(A)** in Figure, representing fortified breast milk feeds assigned with an assumed native protein content of 1 g.dL^−1^ suggests that the recommended daily intake (RDI) for protein can only be met when the milk feed is fortified with Fortifier B and fed at 180 mL.kg^−1^.d^−1^. Yet graph **(B)**, representing fortified breast milk feeds assigned an assumed native protein content of 1.27 g.dL^−1^, suggests the lower RDI for protein can be met when the milk feed is fortified with Fortifier B and fed at 150 mL.kg^−1^.d^−1^ and that protein requirement is exceeded when this feed is fed at 180 mL.kg^−1^.d^−1^. Graph **(C)** suggests that breast milk assigned an assumed native protein content of 1.5 g.dL^−1^ exceeds protein requirement when fortified with either fortifier B or fortifier C and fed at targeted volumes of 180 mL.kg^−1^.d^−1^.

When nutrient composition is the primary consideration for choosing a brand of fortifier, dietitians ranked protein content, osmolality then iron in order of importance (Figure 3). Indeed, of the human milk fortifiers currently available in A&NZ, all of which are used in, and imported from other countries, our survey indicates that the most common fortifier employed by units is that which contains the highest amount of protein, has an acceptable osmolality when added to breast milk and contains iron. When used as directed in 100 dL^−1^ of breast milk, the differences in protein content from that which is ranked first to those ranked second and third total 0.34 and 0.44 g, respectively. Whilst these differences may seem insignificant, they reflect between 27 and 35% of the assumed protein values used to represent the native content of preterm breast milk and the three graphs in Figure 4 demonstrate the relevance of these variations.

The complexity of this issue intensifies when consideration is also given to the variations in assumed values for fat, carbohydrate, energy and micronutrient content of breast milk and the variations in the macro- and micronutrient profiles of milk fortifiers. Collectively, these factors have capacity to result in different estimates of enteral nutrient intakes, underpin and inform different site fortification policies and feeding practices, direct different vitamin and mineral supplement use and misdirect industry formulation of fortifiers, which in turn can ultimately impact the capacity of fortified breast milk and supplement use to appropriately meet the nutrition requirements of preterm infants. In the literature, these variations are inadequately reported (16), making it difficult to interpret and synthesize data, potentially adding to uncertainty of care, increased variation in practice and/or implementation and standardization of inappropriate practices.

### Donor Milk

In recent decades, there has been a groundswell of clinical support for using donor human milk when preterm mother's milk is unavailable or in low supply (50). This is likely due to its demonstrated value in reducing risk of NEC (51) and in promoting feeding tolerance (6) in preterm infants. Earlier concerns that infants grow poorly on donor milk compared to formula have been somewhat allayed by a recent study demonstrating that very low birth weight infants can grow as well with fortified donor milk as they can with formula (52). Our survey shows that when available, donor milk is either pasteurized and sourced from an on- or off-site donor milk bank or is a peer-to-peer donation of unpasteurized milk. Concerns around donor milk pertain to the ethical implications associated with its use, and to ensuring the donation is safe and causes no harm to the donating mother or her infant or to the donor recipient (53). Especially pertinent is ensuring access to support and interventions for mothers and infants to minimize harm to maternal breastfeeding outcomes and to optimize the capacity for a mother to provide sufficient milk for her own infant (53). Equally important are the safety and quality processes underpinning the receipt of donor milk, to ensure donors and the milk itself are screened to reduce hazards associated with communicable and non-communicable disease, lifestyle and diet, medicine and medication use, vaccinations and environmental exposures. Further, the collection, storage and method of processing milk can each introduce their own hazards (53). The justification for using donor milk should be that there is evidence of benefit and that harm can be minimized. Risk assessment and mitigation strategies must be encouraged to minimize risk.

### Probiotics

Controversies exist generally around use of probiotics, largely due to quality and safety concerns in relation to microorganism specification, their numbers, functional properties and presence of contaminants, leading to recommendations for more stringent and mandatory quality control procedures for commercial probiotic products (54). Given this, lack of clinician and researcher confidence in their use in preterm infants is unsurprising, not only because of the safety and efficacy concerns, but also the practical issues associated with their use, such as choice of brand, strain, dosing, form (powder vs. liquid), reconstitution practices, and timing and mode of administration. It has been argued by some, that as a class of intervention, multiple systematic reviews provide evidence of benefit, and therefore probiotics should be incorporated as a standard prophylaxis while good quality research continues to seek to understand the efficacy and effectiveness of single and combined probiotic strains and optimal dosing and duration of administration (55). Others however, are more reticent. A 2020 position paper by the European Society for Paediatric Gastroenterology Hepatology and Nutrition (ESPGHAN) (15) has provided conditional recommendations based mostly on low certainty of evidence, about which specific strains might potentially be used and which should be avoided in the preterm population. This Society has also addressed several safety issues with mitigation strategies that they suggest should be applied to preterm probiotic use. Among the 15 conditional recommendations, there is a requirement when implementing a probiotic product to inform local microbiologists and confirm their ability to routinely detect probiotic bacteraemia/fungaemia with standard culture methods; to use only probiotic products manufactured according to good manufacturing practices to ensure correct strain identity with lack of contamination; to select a strain of probiotic with proven effectiveness and established safety profile; to use similar doses as applied in relevant RCTs; to treat for a duration based on the recipient population and their ongoing risk of diseases; and that use of a single strain or combination of strains be practice-based on positive results from well-conducted RCTs in the clinical setting and that research settings be used to test new strains or combinations of strains.

A systematic review of randomized trials published in 2021, with data spanning 45 years from 1973 and including either adult or pediatric patients, was conducted to determine the efficacy of a limited number of single vs. multi-strain probiotic mixtures, while also accounting for strain and disease specificity (56). Inclusion of studies into this review was limited to probiotics that had efficacy trials with at least one trial using a single strain and at least one RCT of a multi-strain mixture containing a least one matching single strain. Single-strain probiotics that had not been incorporated into multi-strain mixtures were not included. Multi-strain mixtures that had no corresponding efficacy trials from at least one single strain in the mixture were also excluded. For prevention of NEC, 10 randomized controlled trials conducted between 2002 and 2016 were included. The probiotic strains in these trials were either *Bifidobacterium lactis* Bb12 alone (*n* = 3) or *Lactobacillus rhamnosus* GG alone (*n* = 4) and four two-strain mixtures (*n* = 1 RCT each), namely *Bifidobacterium lactis* Bb12 and *Bifidobacterium longum* Bb536, or *Lactobacillus rhamnosus* GG and *Bifidobacterium lactis* Bb12 or *Lactobacillus rhamnosus* GG and *Bifidobacterium lactis* Bb536 or *Lactobacillus rhamnosus* GG and *Bifidobacterium lactis* HN19. The meta-analysis showed that the single strain *Lactobacillus rhamnosus* GG (*RR* = 0.17, 95% CI 0.07, 0.41) was more effective than the two mixtures containing additional strains for prevention of NEC. Notably, whilst duration of dosage was similar, the mean dose in the four trials using *Lactobacillus rhamnosus* GG alone was significantly higher than the mean dose of *Lactobacillus rhamnosus* GG when used in mixtures, perhaps highlighting the importance of repeated multicenter clinical investigations using a single protocol to ensure reproducibility and to increase confidence in the safety and efficacy of probiotic use in the preterm population (57).

When all safety conditions are met, the two conditional recommendations made by ESPGHAN for their potential capacity to reduce NEC stage 2 or 3 (in doses specified in their publication), are use of the single strain *Lactobacillus rhamnosus* GG and use of the combination of *Bifodobacterium Infantis* Bb-02, *Bifidobacterium Lactis* Bb-12 and *Streptococcus Thermophilus* TH-4 (15).

The four brands of probiotics in use across 23 ANZNN NICUs represented in our survey come in the form of capsules/powder and/or drops and differ between brands in the number and type of strains they each contain. Of the four brands reportedly in use, one is a multi-species formulation, containing *Lactobacillus acidophilus, Bifidobacterium infantis* and *Bifidobacterium bifidum*; another contains both *Lactobacillus acidophilus* and *Bifidobacterium bifidus*; and of the remaining two, one contains a single strain, *Lactobacillus rhamnosus* GG and the other, *Bifodobacterium Breve* M-16 V. It is somewhat reassuring that across ANZNN NICU sites, the most recent ANZNN annual report documents the percentage of preterm babies with confirmed NEC at 1.4% of all registrants [*n* = 154/10,494; <28 week: *n* = 96 (8.4%); 28–31 week: *n* = 28 (1.2%)] (58).

### Monitoring Preterm Growth

The universal goal in the nutrition management of preterm infants is to safely provide nutrients to match the rate and composition of weight gain of a normal healthy fetus that remains *in utero* until full-term (59, 60). The appropriateness and feasibility of this growth target has been debated for decades, with EUGR, most commonly defined in the literature as weight <10th percentile at 36 weeks' postnatal age, being identified as an almost universal problem among preterm infants born with weights below 1,500 g (61–67). The history of this poor postnatal growth has been largely due to nutrition deficits exacerbated by an underestimation of preterm nutrient requirements (68–71) and conservative nutrition practices, namely lack of parenteral nutrition or delay in replacing placental nutrition with parenteral nutrition, slow increments in enteral feeding, and frequent and lengthy withholding of feeds due to real or perceived feeding intolerance. More recently, while striving to achieve latest recommended protein and energy intakes (59, 60), concerns about rapid catch up growth and its metabolic implications shifted clinican and research focus to trying to find the right balance between prescribing earlier, more aggressive nutritional support and mitigating any associated metabolic risk (72). In 2016, Rochow and colleagues (73) assisted with this by demonstrating with their analysis of preterm postnatal growth and the Fenton Fetal-Infant Growth Reference, that the physiological diuresis that occurs during the first days of life due to the irreversible contraction of extracellular water space permanently shifts the postnatal growth trajectory by −0.8 z-scores, after which time it tracks parallel to intrauterine curves with growth rates similar to those *in utero*. Last year, Fenton and colleagues (17), also argued that using the arbitrarily nominated <10th percentile at 36–40 weeks corrected age or earlier discharge, as a proxy for EUGR, is inappropriate as it considers weight at a corrected age at one point in time without reference to fetal growth or the birth percentile or the percentile at which birth weight is recovered after adaptation to the extrauterine environment; it does not consider head, length, proportionality or composition of growth, and it does not consider genetic potential. Rather, the degree of change in z score from birth, corrected for the contraction of extracellular water during adaptation to extrauterine life, may be a more relevant indicator of extrauterine growth restriction. Indeed, recent recommendations for primary indicators of mild, moderate and severe malnutrition in preterm infants after the first 2 weeks of life are a decline in weight-for-age z score of 0.8–1.2, >1.2–2, and >2, respectively (74). It is likely that robust preterm growth reference standards that represent the full spectrum of viable preterm birth gestations would be most ideal for assessing adequacy and aberrations of serial preterm growth measurements; it is unfortunate that the few available at this time have serious limitations for monitoring the postnatal growth of infants born extremely preterm (75, 76).

The seven cross-sectional size-at-birth preterm growth charts in use across A&NZ neonatal sites were derived from different datasets and time periods and have diverse features, the most important relating to gender specificity, age accuracy, sample representation across the gestations, generalizability to the global preterm population, harmonization with WHO growth standards, and capacity to calculate z-scores (Table 3). Many of these features have been discussed in various detail elsewhere (16, 77).

To further summarize, the Kitchen et al. (37) dataset is derived from growth measurements taken in the 1970's in the Australian state of Victoria and is older and considerably smaller than the New South Wales dataset used to construct the Beeby charts (38). Neither dataset is completely gender-specific across the gestational ages their data represents and the curves, constructed in completed weeks, do not harmonize with the term WHO Standards and are not accompanied by z-scores. Calculation of change in z-score for assessing the association between growth and neurodevelopment has been found to be superior than the arbitrarily assigned cut-off of <10th percentile and they are useful method for examining and comparing growth rates relative to a growth chart after the early accommodation to extrauterine life (17). Z-scores accompany the gender and country-specific UK-WHO charts and these curves also complement the term WHO Standards; however, the UK curves were constructed in completed weeks on measurements taken mainly from East Anglia (excluding non-white participants) during a similar time period as Beeby's, though from fewer measurements and they are unusual in that they deviate from the seeming convention of 3rd, 10th; 25th, 50th 75th, 90th; and/or 97th centiles.

The meta-analysis conducted by Fenton et al. (40) for the construction of the Fenton 2013 preterm centile charts incorporate in excess of 4 million measurements taken during the period 1991–2007, with 260,000 weight measurements and 150,000 measurements each for length and head circumference taken of infants born <36+6 weeks gestation; almost 23,000 of these weight measurements, and over 9,000 each of these head and length measurements were taken of infants ≤ 27+6 weeks gestation. All data originated from developed countries, including from Australia, Scotland, United States of America, Canada, Germany and Italy, and are represented as actual age (weeks + days) to support growth monitoring in the clinical setting and to harmonize with the World Health Organisation Growth Standard by 10 weeks post term age. The statistical smoothing of the data between the preterm and WHO estimates maintains the integrity of the data from 22 to 36 and at 50 weeks while attempting to account for the intrauterine deviation in growth velocity that occurs during the weeks just prior to, and after, birth; this deviation is likely due to early postnatal fluid shifts, physiological adaptation to extrauterine life as well as alteration in metabolism and nutrition. Reassuringly, studies have shown that preterm infants generally grow approximately parallel to the Fenton preterm growth chart curves (73, 78), in keeping with the expert recommendations for growth rates (59).

The gender-specific International Standards for Newborn Size-at-Birth, originating from a sub-study within the Intergrowth-21st Project (42), were long awaited and anticipated as being the preferred international new-born growth standard for all infants. The intention had been to develop a Standard that would be universal and independent of time, that unlike a growth reference, would not be representative of a given population or region at a given time, and could be used to assess the size of newborn infants, irrespective of ethnicity, locality, socioeconomic status, or health-care provision. The data were collected using standardized measurement techniques from neonates without major congenital abnormalities or ultrasound evidence of fetal growth restriction (FGR) and were born to healthy mothers receiving antenatal care who were without FGR risk factors and living within chosen regions within Brazil, Italy, United States of America, United Kingdom, India, Kenya, China and Omen (42). Notably, the very small dataset arising from the Project's prescriptive inclusion criteria dictated a lower gestational limit of 33 weeks for the centiles (33 week: weight *n* = 51; length: *n* = 50; head circumference: *n* = 50), making the standards of limited use to clinicians wishing to assess growth of infants born at much earlier gestations.

Subsequent to the publication of these standards, the dataset was supplemented with newborn data obtained from infants born without congenital malformations or ultrasound evidence of FGR before birth, to mothers in the Intergrowth-21 consortium whose fetal growth risk factors did not include smoking and/or severe obesity. Two hundred and eighty-five weight measurements and 249 each of length and head circumference measurements were used to construct the gender-specific newborn, weight, length and head circumference reference centiles for gestations 24^+0^-31^+6^ weeks. Values for birthweight and head circumference at 33 weeks' gestation overlapped well on the original International Newborn-Size Standards and values for length harmonized around the 50th centile, but were less complementary at the more extreme curves. The authors of these charts themselves caution that below 28 weeks, the centiles should be interpreted with care, given the small sample size used to generate the curves. Thus, these curves are not optimal for assessing growth of preterm infants born extremely preterm.

An alternative to INTERGROWTH-21st very preterm size-at-birth reference charts could be INTERGROWTH-21st postnatal preterm growth standards (43), however only 28 infants participating in this component of the INTERGROWTH-21st project were born ≤ 33 weeks (12 between 27 and 32 weeks), and the mean gestational age and weight of the preterm infants were 35.5 (SD 1.7) weeks and 2,452 g (519) (Table 4). The developers of these charts claim the power of their study to be equivalent to the power of a cross-sectional study of 3,500 size-at-birth measurements. However, there are several limitations associated with this dataset, not the least of which is the lack of any data below 27^+0^ weeks' gestation, the very small number of preterm infants included between gestations 27 and 32 weeks, the 2-week gap between birth and the subsequent measurement, and the fact that only three measurements were taken for each infant in the first month of life (75). Further, details about the postnatal nutrition received by these infants are not documented and the curves, which represent the data in completed weeks, do not harmonize with the WHO charts until 14 weeks post corrected term.

Comparison of Fenton 2013 reference charts with Intergrowth-21 preterm size-at-birth and the postnatal growth standards (79) has revealed that when using the Intergrowth curves, more infants are classified as small for gestational age (SGA) and fewer are classified with EUGR (defined as <10th percentile; or z-score −1.28) at 36 weeks' gestation or earlier discharge. Logically, given the methodological differences between Fenton and the Intergrowth-21st project, this finding is not unexpected. However, as discussed earlier, arbitrarily using <10th percentile at 36 weeks gestation or earlier discharge as a proxy for EUGR may not be appropriate. Further, the classification of SGA (weight <10th percentile for gestational age) is applied to an infant whose birth weight is lower than the population norm on a growth chart for a particular *birth* gestation; it does necessarily imply pathologic *in utero* growth abnormalities and is not equivalent to the term ‘intrauterine growth restriction’ (IUGR), which is a clinical definition, and when applied to an infant at birth, refers to a rate of *fetal* growth that is less than normal for the growth potential of a specific infant as per the race and gender of the fetus (80, 81). An IUGR infant may have an appropriate birth weight as per gestation, but may have suffered any *in utero* growth deceleration as a consequence of a perinatal insult (80). Notably, poor neonatal growth categorized using Fenton's preterm size-at-birth growth charts have shown stronger associations with long term neurodevelopment than poor growth categorized using the Intergrowth 21st Standards (82).

In March 2019, ANDiN reached a consensus to use Fenton growth charts and accompanying z-scores to monitor the growth of preterm infants up to 50 weeks post-menstrual age, with transition to the WHO growth standard to 2 years of age in both clinical practice and the research setting. A recommendation to this effect was made to the neonatal community by ANDiN at the 2019 Perinatal Society of Australian and New Zealand Congress.

### Calculating Growth Velocity

Although daily weights, weekly length and weekly head circumference are common and recommended measures used in the clinical setting (83), growth velocity is a frequently reported outcome measure in neonatal research and is a useful clinical measure when comparing an infant's rate of growth to the fetal target growth. Calculating actual growth velocity from daily weight measures is labor intensive and commonly, and as our survey demonstrates, and as evidenced around the globe by others (36), clinicians and researchers estimate growth velocity using a variety of mathematical models. These variations in practice make it difficult to compare growth outcomes among preterm nutritional studies and limits the evidence upon which to standardize nutritional care. Patel et al. (36) compared the accuracy of three mathematical methods for estimating average growth velocity with actual growth velocity calculated from daily weight measures of preterm infants and though there was wide variation among the estimates, the exponential model was found to be extremely accurate. ANDiN recommends Patel's exponential model is adopted by all neonatal researchers to calculate and report the growth velocity of extremely (36) and very low birth weight (35) infants.

### The Challenge

Evidence-based medicine is the best approach to reducing clinical uncertainty, yet many of the neonatal nutrition practices that are in wide and accepted use are not supported by strong evidence and these global variations have proven a major limitation to the synthesis of neonatal research data and to progressing universally-adopted, standardized evidence-based practices. It is hoped that this survey might be impetus for other NICU centers to explore the degree to which their own nutrition practices differ from others and reflect on justification and evidence for these practices.

Indeed, it has recently been argued that in order for medicine to advance, a paradigm shift is necessary, where the default, and fairest approach to care might be to randomize patients to the allocation of widely utilized and accepted treatments, but where the evidence base is actually uncertain, so that the chance of receiving the yet to be determined best treatment is unaffected by clinician bias, and where care is delivered along a pre-designed, closely monitored pathway. Informed patients could choose to opt out, rather than in, to the randomized allocation of treatment and trial data could be largely extracted from electronic records and databases (26). Elucidating the role of preterm nutrition and growth on neurodevelopment, metabolic and other important neonatal outcomes might be achieved more successfully and expediently if this strategy could be successfully applied in neonatology. First though, the reporting and data entry of neonatal nutrition and growth outcomes needs to be standardized. To this end, ANDiN concurs with previous recommendations from the StRONNG Checklist (16), though with stated revisions according to more recent literature (Table 5).

**Table 5:** ANDiN consensus for nutrition practice, calculations, and reporting [adapted from (16)].

Nutrition calculations (33, 84)	**Enteral:** - Carbohydrate: 4.0 kcal (16.7 kJ).g^−1^ - Protein: 4.0 kcal (16.7 kJ).g^−1^ - Fat: 9.0 kcal (37.7 kJ).g^−1^ **Parenteral:** - Glucose: (anhydrous) 3.8 kcal (15.9 kJ.g^−1^); (monohydrous) 3.4 kcal (14.23 kJ.g^−1^); (refer to product information) - Nitrogen: (refer to amino acid product information) - Conversion of nitrogen to protein equivalence (refer to amino acid product information) - Protein: 3.8 kcal (15.9 kJ).g^−1^ (refer to amino acid product information) - SMOF Lipid (20% emulsion): 10 kcal (41.8 kJ).g^−1^ lipid; when using SMOF lipid emulsion with vitamins added to emulsion, refer to lipid emulsion product information and check composition, amount of vitamins added and calculations with supplier/your pharmacy.
Preterm human milk composition estimate (1, 31)	**Macronutrients** - Protein: 1.27 g.dL^−1^ - Fat: 3.46 g.dL^−1^ - Lactose: 6.15 g.dL^−1^ - Carbohydrate: 7.34 g.dL^−1^ Micronutrients: Koletzko et al. (31) (Appendix 2)
Growth charts (40, 85)	**Preterm** - Fenton 2013 and z-scores up to 50 weeks PMA; then WHO growth Standard to 2 years of age and z-scores. Term - WHO growth Standard 0–2 year
Growth velocity (35, 36)	• Patel's exponential model
	$\frac{\left[1,000\times ln\left(W_{\text{n}}-W_{1}\right)\right]}{\left(D_{\text{n}}-D_{1}\right)}$
	Where W is weight in grams, D is day, 1 indicates the beginning of the time interval and n is the end of the time interval, in days.
Growth assessment (17)	- Birth z-scores for weight, length and head circumference - Δ z-score from birth to 4 weeks, 36 weeks, 40 weeks, 44 weeks, 50 weeks, and discharge - Δ z-score from regaining birthweight to 4 weeks, 36 weeks, 40 weeks, 44 weeks, 50 weeks and discharge - Consideration of early contraction of extracellular fluid and adaptation to extrauterine life - Consideration of different growth patterns of the fetus, preterm and newborn infant between 37 and 42 weeks of life.
Donor milk (53)	Pasteurized donor milk, where there is evidence of benefit; and harm can be minimized. *Peer-to-peer milk donation is also practised in NZ*.
Food thickener	• Recommend caution in using thickening agents for preterm and term infants (86–88); Recommend ascertaining amount of thickening agent per 100 g of powdered product; note that manufacturers may recommend adding thickening agent to breast milk in amounts that exceed those permitted in infant formula products, under Australian legislation (89).

## Conclusion

Whilst there has been some evidence of increased standardization of nutritional practice across A&NZ neonatal units in the past decade, some variation continues in sufficient magnitude to impact nutrition interventions and neonatal outcomes. Nutrition surveys, together with nutritional audit and well-designed clinical trials have potential to inform nutrition policies and standardized feeding practices and to improve neonatal outcomes (90). Partnering with, and adequately resourcing and utilizing the support and skill-set of neonatal dietitians to standardize practice and reporting and collaborate on randomized trials and innovative, comparative treatment research, may help to shift the paradigm and produce quality, evidence-based standardized nutrition practice that removes uncertainty of care and improves neonatal outcomes.

## Data Availability Statement

The raw data supporting the conclusions of this article will be made available by the authors, without undue reservation.

## Ethics Statement

The studies involving human participants were reviewed and approved by Auckland District Health Board Research Review Committee ID A+8146. The patients/participants provided their written informed consent to participate in this study.

## Author Contributions

BC and GM conceived the survey concept and drafted the manuscript. BC obtained ethical approval. All authors commented on and approved the final manuscript, participated in drafting the survey, agreement with the manuscript and declare that the content has not been published elsewhere.

## Conflict of Interest

The authors declare that the research was conducted in the absence of any commercial or financial relationships that could be construed as a potential conflict of interest.

## Publisher's Note

All claims expressed in this article are solely those of the authors and do not necessarily represent those of their affiliated organizations, or those of the publisher, the editors and the reviewers. Any product that may be evaluated in this article, or claim that may be made by its manufacturer, is not guaranteed or endorsed by the publisher.
